# Accuracy of Electronic Health Record–Documented Aspirin for Primary Prevention in Adult Outpatients

**DOI:** 10.1001/jamanetworkopen.2023.26237

**Published:** 2023-07-28

**Authors:** Naina Chipalkatti, Geoffrey D. Barnes, Adam Davie, Jennifer J. Griggs, Molly Harrod, Christine Medaugh, Jordan K. Schaefer

**Affiliations:** 1 Department of Internal Medicine, University of Michigan, Ann Arbor; 2 Division of Cardiovascular Medicine, Department of Internal Medicine, University of Michigan, Ann Arbor; 3 Institute for Healthcare Policy and Innovation, Ann Arbor, Michigan; 4 Division of Hematology/Oncology, Department of Internal Medicine, University of Michigan, Ann Arbor; 5 Department of Health Management and Policy, University of Michigan, Ann Arbor; 6 Center for Clinical Management Research, VA Ann Arbor Healthcare System, Ann Arbor, Michigan; 7 Department of Family Medicine, University of Michigan, Ann Arbor

## Abstract

This quality improvement study examines the accuracy of electronic health record (EHR) documentation of aspirin use for primary prevention of atherosclerotic cardiovascular disease in adult outpatients.

## Introduction

Aspirin is generally considered safe and is widely^1^ used for the primary prevention of atherosclerotic cardiovascular disease. However, for many patients taking this medication, the associated risks outweigh the benefits. Overuse of aspirin has been a longstanding issue^2^ and is increasingly recognized with the publication of recent guidelines^3,4^ that recommend a reduced role for aspirin in primary prevention. Implementing guidelines in clinical practice may be challenging given the variable capture of aspirin use in the electronic health record (EHR).^5^ This study seeks to determine the accuracy of EHR documentation of aspirin use.

## Methods

In this quality improvement study, adults older than 40 years with a primary care physician at 1 of 2 community-based primary care practices were sent an electronic survey through the EHR. The project was determined to be not regulated by the University of Michigan institutional review board, and written consent was not required as part of a quality improvement project, in accordance with 45 CFR §46. This study follows the Standards for Quality Improvement Reporting Excellence (SQUIRE) reporting guideline.

Patients with a documented indication for aspirin other than primary prevention of cardiovascular disease were excluded. The survey was developed with input from numerous stakeholders, including 20 clinicians and 5 patient advisors. The survey asked patients about their aspirin use and medical history. It was sent once with no follow-up messages. Responses were collected from February 15, 2022, through March 15, 2022. χ^2^ tests were used to compare respondents and nonrespondents. A 2-sided *P* < .05 was considered statistically significant. Data were analyzed in Excel software version 2305 (Microsoft) and Stata statistical software version 17.0 (StataCorp).

## Results

Of 1460 patients, 668 (45.7%) completed the survey (282 men [42.2%]; mean [SD] age, 62.3 [10.2] years). Twenty-two respondents were excluded for reporting secondary prevention aspirin use. Respondents were similar to nonrespondents with regard to sex, body mass index, and aspirin use as documented in the EHR. However, respondents were more often White (586 of 688 respondents [87.7%] vs 607 of 792 nonrespondents [76.6%]; χ^2^_3_ = 30.0; *P* < .001) and older (mean [SD] age. 62.3 [10.2] years vs 59.2 [11.5] years; mean difference, 3.1 years [95% CI, 2.0-4.2 years]; *t*_1458_ = 5.40; *P* < .001) than nonrespondents.

Among 668 respondents, 497 (74.4%) did not take aspirin most days of the week (Figure). For respondents denying aspirin use, the EHR was accurate in 429 of 497 cases (specificity, 86.3%) (Table). Overall accuracy (calculated as [true-positives + true-negatives] / the total) was 85.3%. If aspirin was not documented in the EHR, that matched the patient report for 429 of 456 cases (94.1%). A total of 149 of 668 respondents (22.3%) reported regular primary prevention aspirin use. Of them, 122 of 149 (sensitivity, 81.9%) had aspirin correctly documented in the EHR. If aspirin was documented in the EHR, it matched the patient report for 122 of 190 cases (64.2%). Of 149 primary prevention aspirin users, 130 (87.2%) had taken aspirin for 11 to 14 days in the past 2 weeks, 130 (87.2%) were taking a dose of 81 mg per day or less, and 105 (70.5%) had been taking aspirin for more than 5 years.

**Figure.**
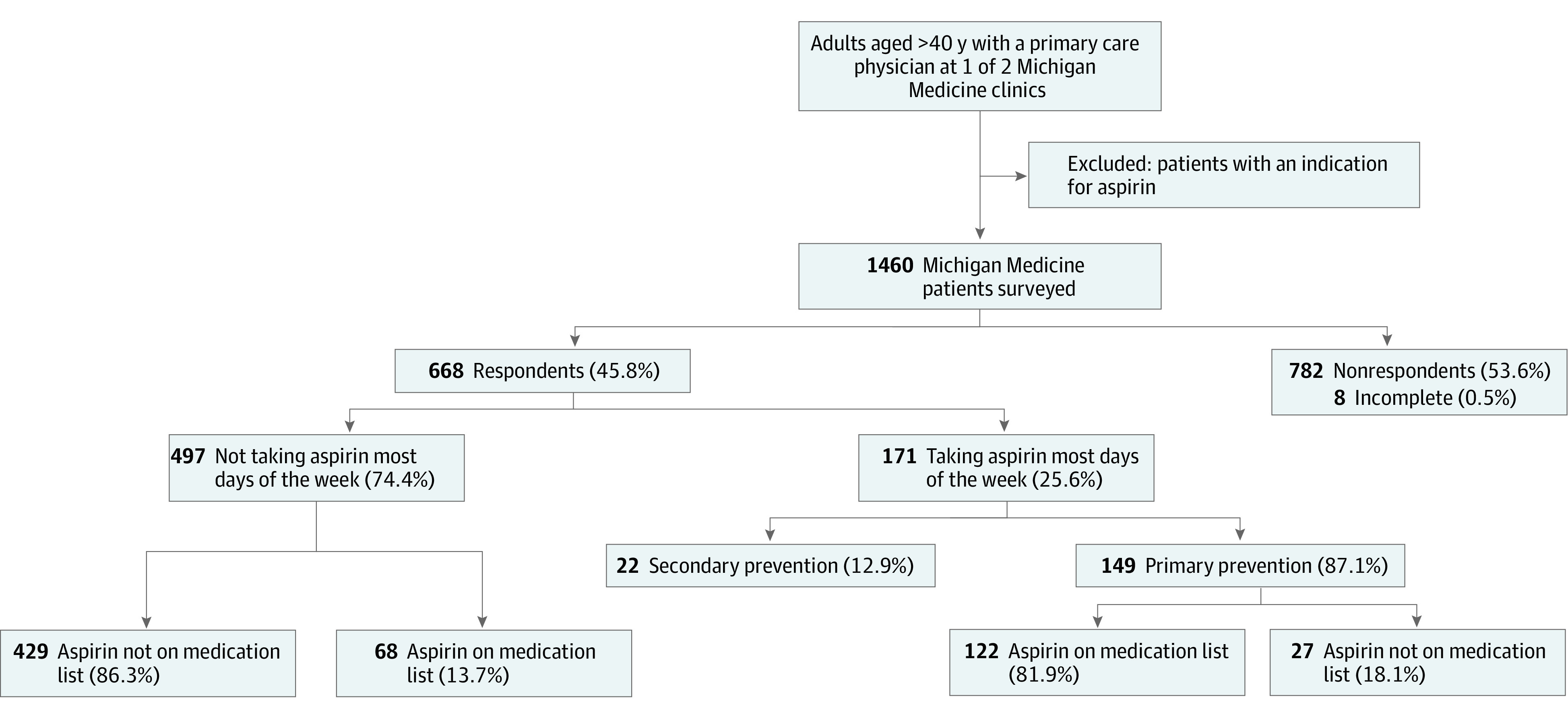
Flowchart Depicting Survey Responses

**Table.** Accuracy of the Electronic Health Record for Recording Aspirin Use^a^

Variable	Survey responses, No.		
	Aspirin use reported	No aspirin use reported	Total
Aspirin on medication list	122	68	190
Aspirin not on medication list	27	429	456
Total	149	497	646

^a^ Sensitivity was 81.9% (95% CI, 75.7%-88.1%), specificity was 86.3% (95% CI, 83.3%-89.3%), the positive predictive value was 64.2% (95% CI, 57.4%-71.0%), and the negative predictive value was 94.1% (95% CI, 91.9%-96.3%).

## Discussion

In this quality improvement study of adults older than 40 years in primary care clinics affiliated with an academic medical center, overall, the EHR correctly identified whether patients were taking aspirin with 85.3% accuracy vs the patient report (Table). It is reassuring that clinicians can accurately identify patients taking aspirin for primary prevention in most instances.

Given the persistence of aspirin overutilization, active deprescribing interventions are necessary.^6^ On the basis of the findings of this study, efforts can reasonably start with patients who list it as one of their medications. A more comprehensive strategy may also involve improving the reconciliation process for over-the-counter medications.

Limitations of this study include those inherent to a cross-sectional design, including the potential for response bias. Aspirin use and health status may have changed between the last clinical encounter and survey response, resulting in misclassification. In addition, this study evaluated 2 Midwestern, university-affiliated clinics. Further studies are needed to evaluate the medication list accuracy of aspirin in other settings.

## Notes

Supplement.
Data Sharing Statement
