# Supplementary material for: Accuracy of Electronic Health Record–Documented Aspirin for Primary Prevention in Adult Outpatients
Source: JAMA Netw Open. 2023 Jul 28;6(7):e2326237. doi: 10.1001/jamanetworkopen.2023.26237 (PMC10383008; doi:10.1001/jamanetworkopen.2023.26237)
Supplement: Supplement. — Data Sharing Statement [file jamanetwopen-e2326237-s001.pdf]

Data Sharing Statement

Chipalkatti. Accuracy of Electronic Health Record–Documented Aspirin for Primary Prevention in Adult Outpatients. *JAMA Netw Open*. Published July 28, 2023. doi:10.1001/jamanetworkopen.2023.26237

Data
Data available: No
